# Biomimetic Model of Contractile Cardiac Tissue with Endothelial Networks Stabilized by Adipose-Derived Stromal/Stem Cells

**DOI:** 10.1038/s41598-020-65064-3

**Published:** 2020-05-20

**Authors:** Justin Morrissette-McAlmon, Brian Ginn, Sarah Somers, Takuma Fukunishi, Chanon Thanitcul, Alexandra Rindone, Narutoshi Hibino, Leslie Tung, Hai-Quan Mao, Warren Grayson

**Affiliations:** 1 Translational Tissue Engineering Center, Johns Hopkins University School of Medicine, Baltimore, MD USA; 2 Department of Biomedical Engineering, Johns Hopkins University School of Medicine, Baltimore, MD USA; 3 Department of Material Sciences & Engineering, Johns Hopkins University, School of Engineering, Baltimore, MD USA; 4 Institute for NanoBioTechnology (INBT), Johns Hopkins University School of Engineering, Baltimore, MD USA; 5 Department of Surgery & Cardiac Surgery, Johns Hopkins University School of Medicine, Baltimore, MD USA

**Keywords:** Bioinspired materials, Regenerative medicine, Tissue engineering

## Abstract

Cardiac tissue engineering strategies have the potential to regenerate functional myocardium following myocardial infarction. In this study, we utilized novel electrospun fibrin microfiber sheets of different stiffnesses (50.0 ± 11.2 kPa and 90.0 ± 16.4 kPa) to engineer biomimetic models of vascularized cardiac tissues. We characterized tissue assembly, electrophysiology, and contractility of neonatal rat ventricular cardiomyocytes (NRVCMs) cultured on these sheets. NRVCMs cultured on the softer substrates displayed higher conduction velocities (CVs) and improved electrophysiological properties. Human umbilical vein endothelial cells (HUVECs) formed dense networks on the sheets when co-cultured with human adipose-derived stem/stromal cells (hASCs). To achieve vascularized cardiac tissues, we tested various tri-culture protocols of NRVCM:hASC:HUVEC and found that a ratio of 1,500,000:37,500:150,000 cells/cm^2^ enabled the formation of robust endothelial networks while retaining statistically identical electrophysiological characteristics to NRVCM-only cultures. Tri-cultures at this ratio on 90 kPa substrates exhibited average CVs of 14 ± 0.6 cm/s, Action Potential Duration (APD)80 and APD30 of 152 ± 11 ms and 71 ± 6 ms, respectively, and maximum capture rate (MCR) of 3.9 ± 0.7 Hz. These data indicate the significant potential of generating densely packed endothelial networks together with electrically integrated cardiac cells *in vitro* as a physiologic 3D cardiac model.

## Introduction

Heart disease is one of the leading causes of death in the Western world^1^. Coronary heart disease is the most common form of heart disease. According to the Center for Disease Control (CDC), there are nearly 790,000 infarcts that occur annually in the United States (U.S.), with approximately 500,000 of those being first time myocardial infarction (MI)^1^. During MI, occlusion of the coronary arteries causes apoptosis and necrosis of cardiomyocytes which, due to the limited endogenous regenerative potential of the myocardium, ultimately results in a fibrotic response, reduced cardiac output, and potential ventricular wall thinning^2^. Cardiac tissue engineering is a promising approach to repair the infarcted area and restore lost functionality. Several strategies have been used to regenerate the damaged myocardium. These range from direct injections of cells suspended in saline to biomaterial-based strategies^3–5^. However, due to the complex architecture of the native myocardium, combined cell-biomaterial-based strategies are generally considered to be quite promising to guide regeneration of fully functional tissues. Key features of engineered cardiac scaffolds that may be controlled to maximize the regenerative response include alignment topography, mechanical compliance, biodegradability, flexibility, and the ability to integrate with the native myocardium^6^.

Fibrin is an attractive biomaterial substrate for engineering vascularized cardiac grafts due to its mechanical characteristics and its ability to promote both cell survival and angiogenesis^7–9^. Fibrin-based scaffolds exhibit strain stiffening behavior similar to that displayed by the myocardium^10^ and have the ability to undergo remodeling and degradation as the cells secrete their own extracellular matrix (ECM) over time. Fibrin has been shown to stimulate the secretion of extracellular matrix^9^. Recently, fibrin has been electrospun into microfibers with diameters of approximately 10 μm. The electrospun fibrin microfibers offer tunable, biomimetic mechanical properties. The reported moduli of native myocardium are 10–20 kPa in the transverse direction (orthogonal to cardiomyocyte alignment) and 40–50 kPa in the longitudinal direction^11–13^). Substrate mechanics influences cell maturation, action potential duration, contractility^14–16^, gene expression, and twitch force^17,18^. Electrospun microfibers can also provide alignment cues, flexible geometry, and mimic the structure of the native ECM^19^.

Contraction of the myocardium is enabled by tightly packed interconnected cardiomyocytes^20^. The high metabolic demands of the cardiomyocytes are supported by densely packed vasculature (endothelial cells supported by vascular mural cells)^21^. Likewise, the survival of tissue engineered grafts relies on the development of interconnected capillary networks that can anastomose quickly with the host vasculature upon implantation^22,23^. Therefore, the objective of these studies was to optimize the *in vitro* conditions that enabled us to engineer a contractile biomimetic myocardial grafts with dense endothelial network assembly. To accomplish this goal, we worked with electrospun fibrin microfiber sheets of two different stiffnesses that correlated with physiologic and pathophysiologic myocardial tissues. We used these to test the effect of elastic modulus on tissue assembly as prior research suggested that the softer substrate would provide increased functionality compared to a stiffer substrate^18,24,25^. To establish the vascular network within a contractile tissue, we incorporated human adipose-derived stromal/stem cells (hASCs) and human umbilical vein endothelial cells (HUVECs) with electrically excitable neonatal rat ventricular cardiomyocytes (NRVCMs). In a previous publication, we have demonstrated that hASCs promoted robust vascularization in co-culture with HUVECs. We assessed the contractile and morphologic properties of the resulting *in vitro* tissue.

## Results

**Figure 1:**
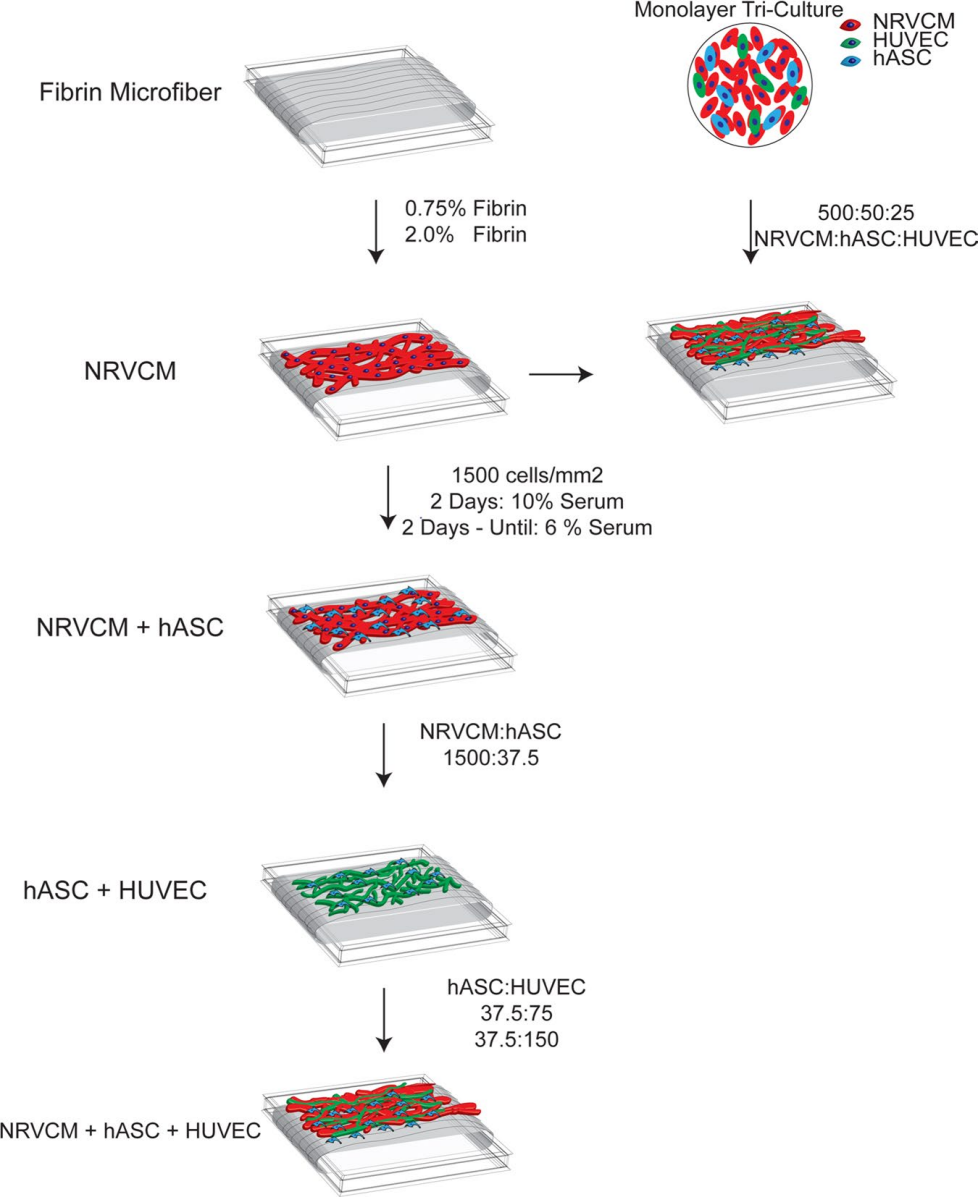
Schematic of experimental design. Fibrin microfiber sheets at two concentrations (0.75% and 2.0% fibrin) were fabricated and characterized. NRVCMs were cultured on fibrin microfiber sheets at different seeding densities to determine the appropriate culture conditions. We initially attempted to use similar cell culture ratios as in previous monolayer studies for the tri-cultures. Since this was unsuccessful, we adopted a step-wise co-culture process. Co-cultures of NRVCMs and hASCs were performed to determine the maximum concentration of hASCs. NRVCMs and hASCs co-cultures could be optically mapped at the ratio of 1500:37.5:0. Vessel development was characterized using co-cultures of hASCs and HUVECs. The best vascular networks were obtained at hASC:HUVEC ratios of 0:37.5:75 and 0:37.5:150. This information resulted in the tri-culture conditions used for the *in vitro* graft.

The overall flow of data in these experiments is depicted in Fig. 1. We characterized the physical properties of the fibrin microfiber sheets used for these studies and then evaluated the growth of NRVCM only, hASC and HUVEC co-cultures, and tri-cultures on these scaffolds. Through the process, we determined the appropriate cell seeding densities, medium compositions, and growth periods required to enable the formation of robust, contractile, vascularized cardiac grafts.

### Fibrin Microfiber Sheets Development and Characterization

**Figure 2:**
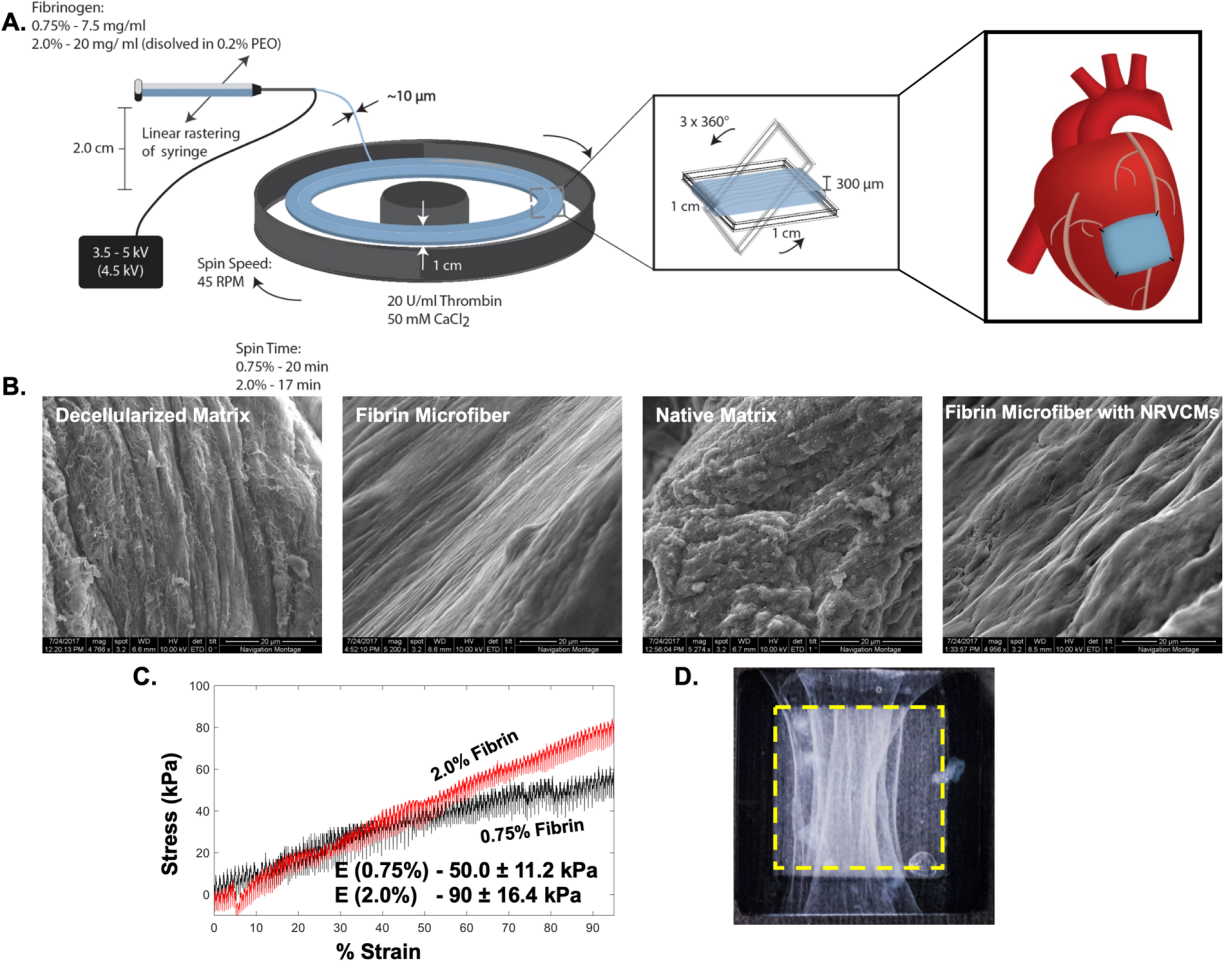
Fibrin microfiber sheet development and characterization (**A**) Schematic illustrating the electrospinning process used to fabricate fibrin microfiber sheets. (**B**) Representative SEM images comparing the decellularized or native myocardium to acellular or NRVCM-seeded fibrin microfiber sheets. (C) Stress strain curve of bulk fibrin microfiber sheets depicting the Young’s Modulus of each concentration of fibrin. (**D**) Representative image of 1 cm × 1 cm fibrin microfiber sheets on mylar frame. Yellow dashed line indicates inner edges of the mylar frame and the boundaries of the scaffold.

Fibrin microfibers were generated through the electrospinning process to form sheets (Fig. 2A,D). SEM images were used to compare the structure of the fibrin microfiber sheets to the native myocardium. The acellular fibrin microfiber sheets exhibited similar alignment and topography as the decellularized myocardium. In addition, there were regions of the NRVCM-seeded microfiber sheets, which showed a similar architecture, compared to the native myocardium (Fig. 2B). In addition, the fibrin microfiber sheets were analyzed for thickness through SEM imaging and found to be roughly near 300 μm. Tensile testing demonstrated the elasticity of 0.75% and 2.0% fibrin microfiber sheets and the resulting stress-strain curves were used to determine their tensile moduli of 50.0 ± 11.2 (n = 3) and 90.0 ± 16.4 kPa (n = 3), respectively (Fig. 2C).

### NRVCM Cultures on Fibrin Microfiber Sheets

**Figure 3:**
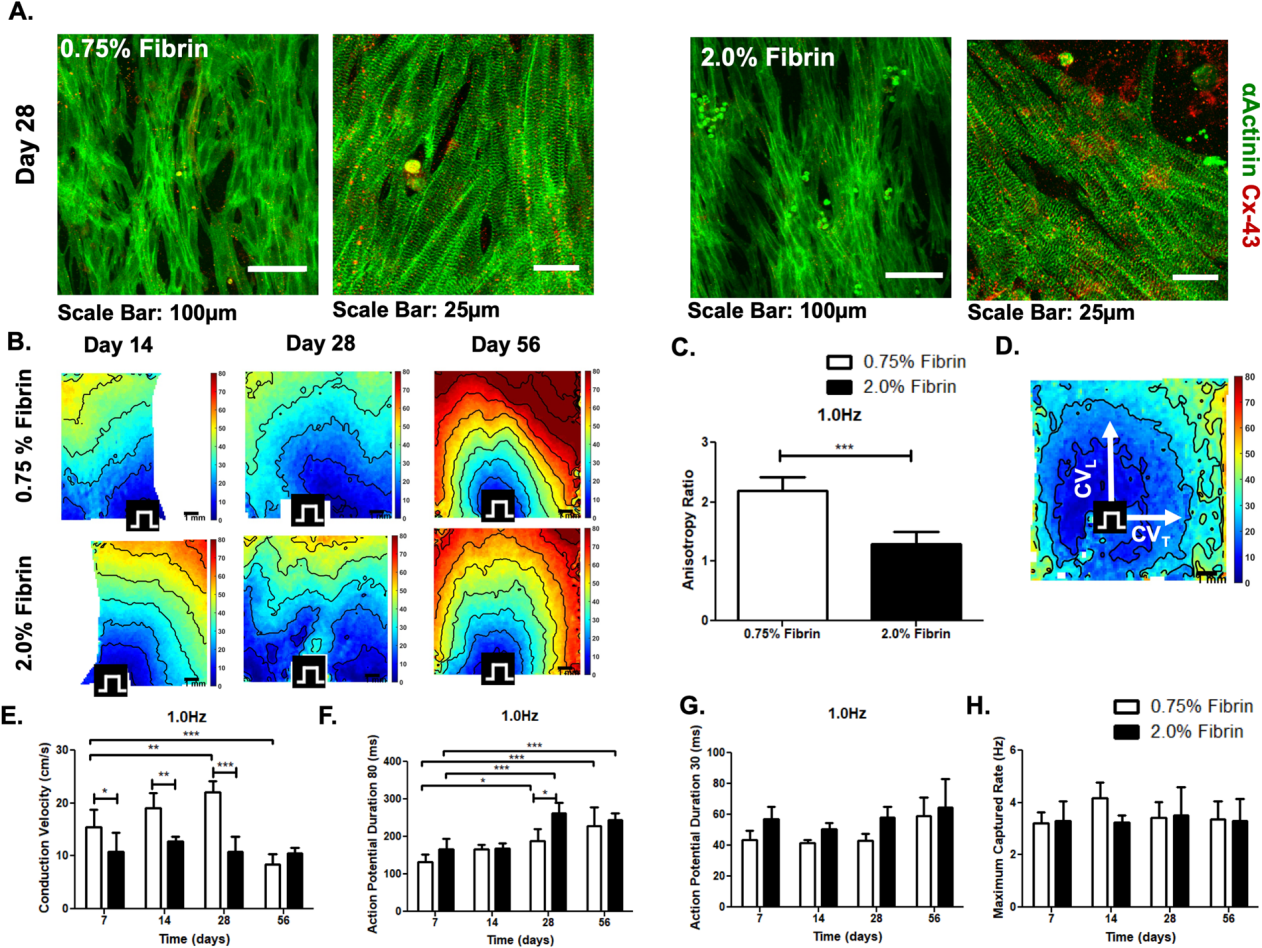
Morphological and Electrophysiological characterization cardiomyocytes on fibrin microfiber sheets. (**A**) Immunofluorescent staining of α-Actinin (green) and Connexin-43 (red) on both 0.75% and 2.0% fibrin microfiber sheets. (**B**) Representative time course (14, 28, and 56 days) activation maps of selected of 0.75% and 2.0% fibrin microfiber cardiomyocyte only group. (**C**) Anisotropy ratio of 0.75% and 2.0% fibrin microfibers at 1.0 Hz pacing rate after 2 weeks of culture. (**D**) Representative activation map of 0.75% fibrin microfiber pacing from the center at 1.0 Hz pacing rate. E-H) Comparison of conduction velocity (**E**), APD_80_ (**F**) APD_30_ (**G**) and maximum capture rates (**H**) of 0.75% and 2.0% fibrin microfibers (n = 4-9). *p < 0.05; **p < 0.01; ***p < 0.001.

NRVCMs used in this study were 73% α-Actinin positive (Fig. S1) and were cultured on both 0.75% and 2.0% fibrin microfiber sheets elongated and aligned on the substrate (Fig. 2A). Data from the PicoGreen DNA assay demonstrated similar DNA content in both groups at 1, 7, and 14 days. Both groups exhibited similar cell viability at Day 14 with calcein AM (live) and ethidium bromide (dead) stain (Fig. S2). Cells grown on both the 0.75% and 2.0% fibrin microfibers were optically mapped at 7, 14, 28, and 56 days. The electrical wave fronts for the NRVCMs exhibited smooth propagations across the field of view when point paced in both groups. We noted that, at early time-points, the cardiomyocytes cultured on the 0.75% fibrin sheets exhibited slightly higher conduction velocities than those on 2.0% fibrin sheets. Longitudinal conduction velocities peaked at 22 ± 2.1 cm/s (n = 3) on day 28 in the 0.75% fibrin group and at 13 ± 0.9 cm/s (n = 3) on day 14 in 2.0% fibrin when samples were paced at 1 Hz (Fig. 3B,E). There was a statistically significant increase in conduction velocity between 0.75% fibers from Day 7 to Day 21. Similar trends in conduction velocity were also noted across other pacing rates from 0.5 Hz to 2.0 Hz (Fig. S3A). The average maximum capture rate peaked at 4.2 ± 0.6 Hz on the 0.75% fibrin microfibers and 3.5 ± 1.0 Hz on the 2.0% fibrin microfibers, although there were no statistical differences among the different time points (Fig. 3H).

The fibers were paced from the center to assess anisotropy and the 0.75% fibrin fibers exhibited an anisotropy ratio of 2.2 (Fig. 3C,D). Action potential durations (APD) at both 80% repolarization (APD_80_) and 30% repolarization (APD_30_) increased over time in both the 0.75% and 2.0% fibrin concentrations. APD_80_ at 1 Hz pacing increased from 133 ± 19 ms (n = 5) at day 7 to 229 ± 51 ms (n = 8) at day 56 in the 0.75% fibrin group (Fig. 3F). A similar trend was noted with the 2.0% fibrin fibers. APD_30_ increased from 43.6 ± 6.0 ms (n = 5) at day 7 to 59.0 ± 12.0 (n = 8) at day 56 in the 0.75% fibrin group when paced at 1 Hz (Fig. 3G). Similar trends were noted for 80% and 30% APD repolarization when paced at 0.5, 1.5 and 2.0 Hz (Fig. S3B,C). The normalized triangulation [Normalized Triangulation = Action Potential Duration (APD)_80_-APD_30_ /(APD)_80_], which is a measure of repolarization rates, was similar amongst all groups with no statistical significance between pacing rates (Fig. S3D). Maximum capture rate for the 0.75% Fibrin microfibers was achieved at 14 days with 4.2 ± 0.6 Hz (n = 6), while the 2.0% Fibrin was 3.5 ± 1.1 Hz (n = 5) at 28 Days.

**Figure 4:**
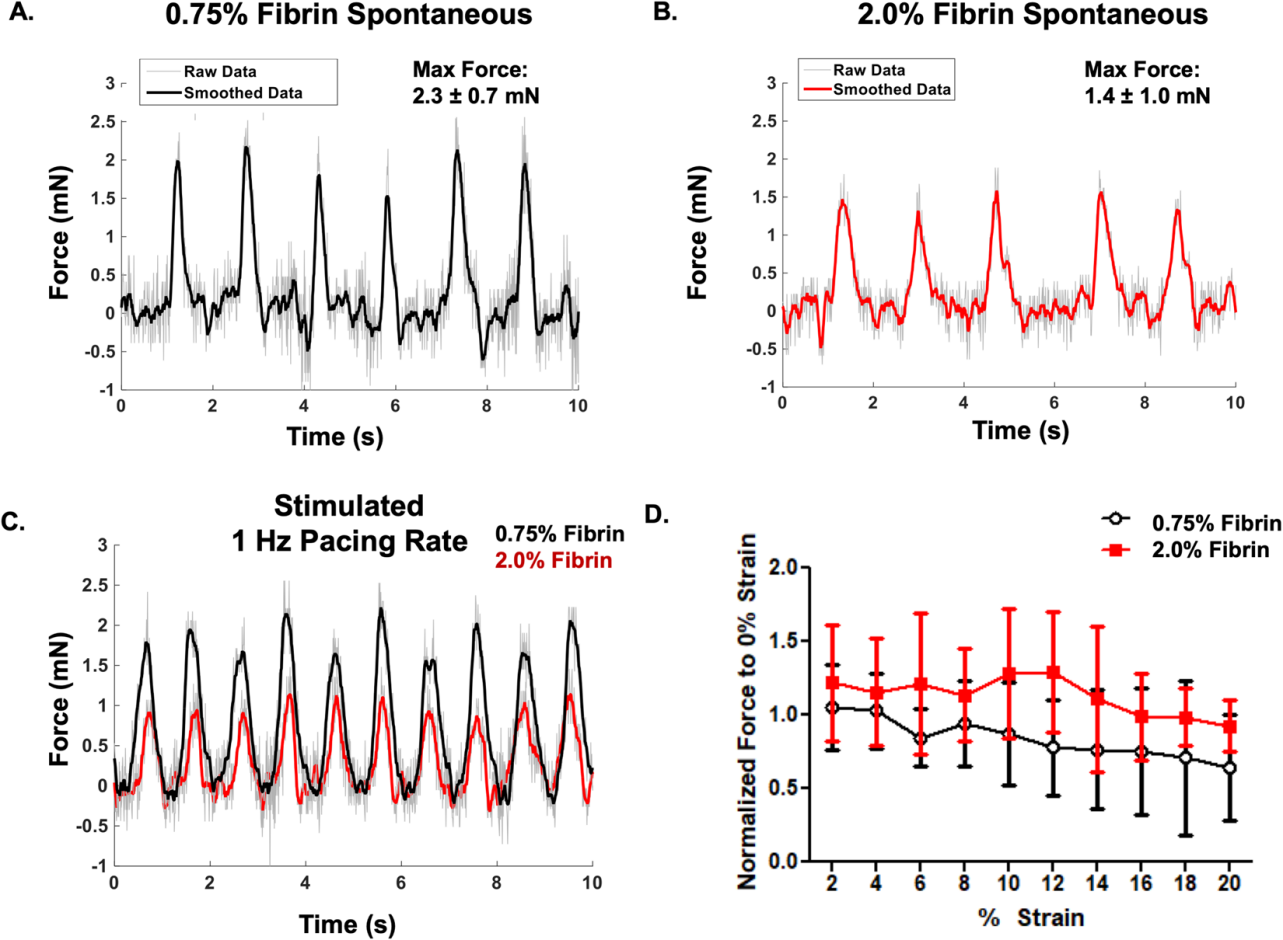
Force of Contraction measurements of Fibrin microfiber sheets. (**A**) Representative spontaneous force trace measurement of 0.75% fibrin concentration microfibers. (**B**) Representative spontaneous force trace measurement of 2.0% fibrin concentration microfibers. (**C**) Representative stimulated force of contraction trace of both 0.75% (black) and 2.0%(red) fibrin microfibers paced at 1 Hz. (**D**) Force length relationship of fibrin microfibers up to 20% strain.

NRVCM-seeded fibrin sheets were spontaneously contractile. At day 14, cardiomyocytes generated 2.3 ± 0.7 mN of force (at frequencies of 0.4 ± 0.3 Hz) and 1.4 ± 1.0 mN of force (at frequency of 0.5 ± 0.4 Hz) on 0.75% and 2.0% fibrin microfiber sheets, respectively (Fig. 4A,B). When stimulated at 1 Hz, the forces of contraction were 1.8 ± 0.4 mN and 0.9 ± 0.5 mN on the 0.75% and 2.0% fibers, respectively (Fig. 4C). Constructs could be paced up to 3 Hz but typically lost capture at higher pacing rates. Fibers were strained up to 20% at 1 Hz pacing to generate force-length relationship data. However, no clearly discernable maxima were observed for cells grown on either substrate (Fig. 4D).

### Tri-Cultures of NRVCM:hASC:HUVEC on Fibrin Microfiber Sheets

To engineer vascularized cardiac patches, hASCs and HUVECs were cultured together with the NRVCMs. We utilized our published culture protocol established for cells grown on plastic coverslips in which the optimal NRVCM:hASC:HUVEC ratio was 500:50:25 (Fig. 1)^26^. Therefore, maintaining the NRVCM seeding density at 1500 cells/mm^2^, we first tested the equivalent ratio (1500:150:75) as well as an additional ratio in which the concentrations of hASCs and HUVECs were further reduced (1500:75:37.5). At these ratios, the electrophysiological properties of the cells were significantly impaired and the vascular networks were not considered sufficiently extensive or interconnected (Fig. S4).

**Figure 5:**
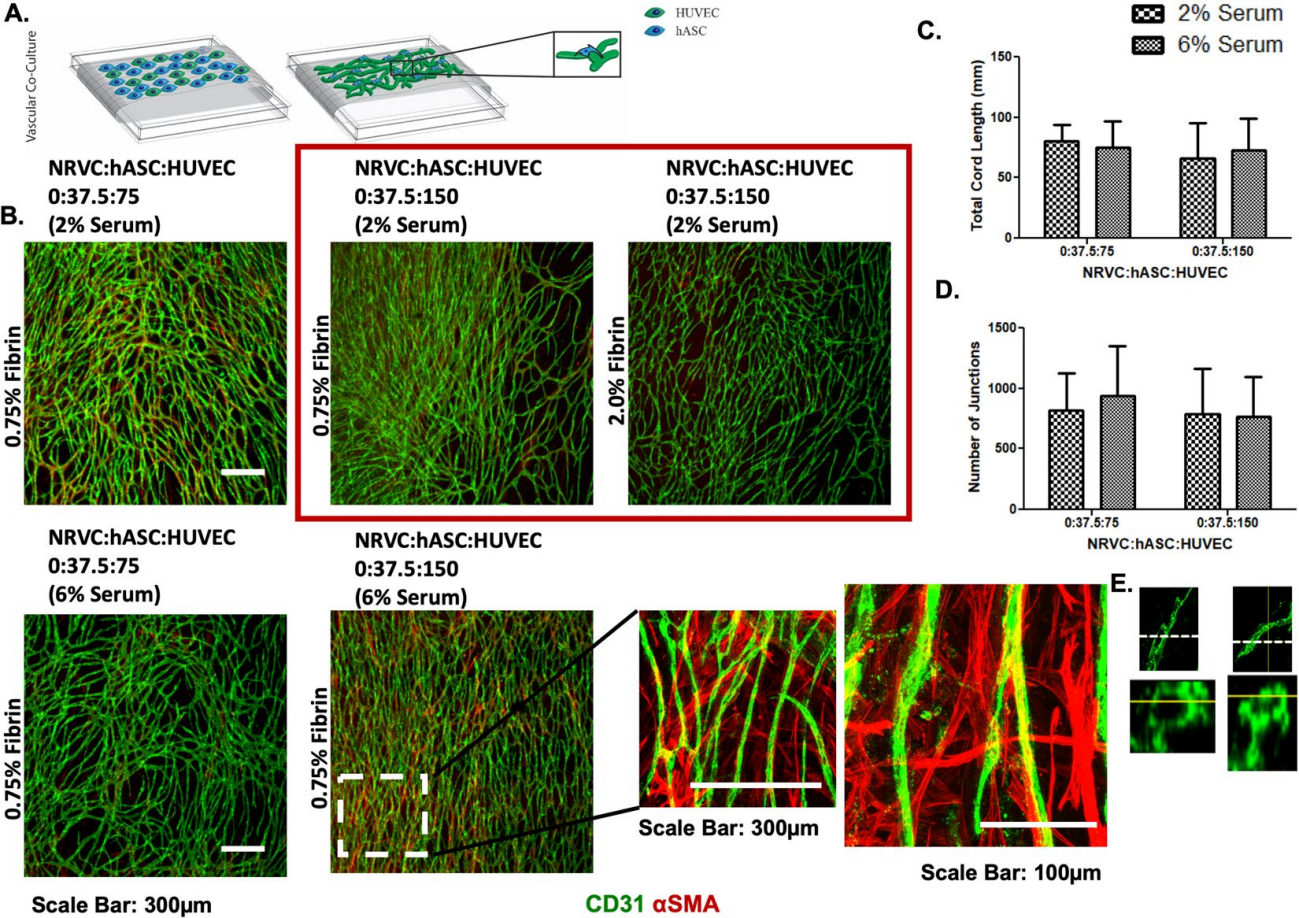
Characterization of vessel development on fibrin microfiber sheets. (**A**) Schematic of vessel development on fibrin microfibers. (**B**) Immunostaining of vessel assembly after several 7 days of culture (**C**) Vessel length of assembled endothelial networks with both 2% and 6% serum (**D**) Interconnectivity of assembled vasculature with both 2% and 6% serum (**E**) Orthogonal view of immunostained vessel on fibrin microfiber.

**Figure 6:**
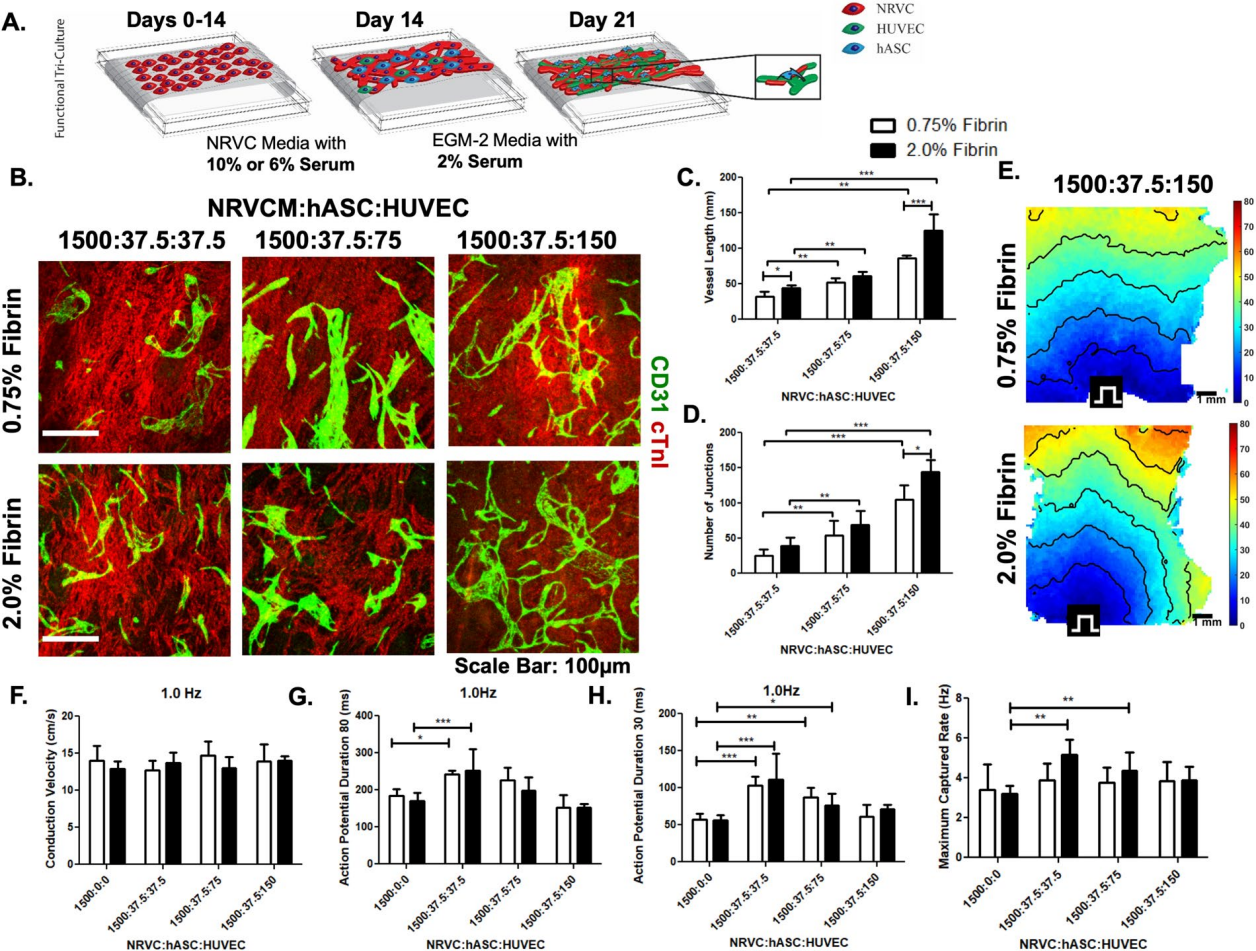
Engineered functional cardiac graft with endothelial networks. (**A**) Schematic showing the additional seeding of supporting cells (hASC and HUVECs) types after 14 days of CM culture. (**B**) Immunofluorescent staining of CD31/PECAM-1 (green) tube-like structures along with cardiac Troponin I (red) on both the 0.75% and 2.0% fibrin microfibers sheets with cell concentrations ranging from 1500:37.5:37.5-1500:37.5:150. (**C**) Comparison of vessel length between titered endothelial cells in tri-culture (**D**) Comparison of the interconnectivity of vessel structures developed in tri-cultures. (**E**) Representative activation maps of both 0.75% and 2.0% fibrin microfiber sheets with tri-cultures at the ratio of 1500:37.5:150 F) Conduction velocity of tri-cultures compared to NRVCM only cultures in both 0.75% and 2.0% fibrin microfibers (**G**) Comparison of Action Potential Duration 80 and (**H**) Action Potential Duration 30 of both 0.75% and 2.0% fibrin microfibers (**I**) Maximum capturerates comparing both fiber stiffness and ratios of NRVCM:hASC:HUVECs. p < 0.05; **p < 0.01; ***p < 0.001.

To address this, we followed a step-wise approach to improve the system. (Step 1) We co-cultured NRVCMs and hASCs in the absence of HUVECs and found that 1500:37.5:0 was the highest ratio of hASCs that could be co-cultured with NRVCMs without producing erratic conductions. However, electrophysiological properties such as conduction velocity and maximum capture rate of the co-cultures at this ratio were significantly inhibited compared to NRVCM only cultures. The conduction velocity was 10.2 ± 0.5 cm/s (0.5 Hz), 9.8 ± 1.4 cm/s (1.0 Hz), 9.2 ± 0.9 cm/s (1.5 Hz), and 8.7 ± 1.0 cm/s (2.0 Hz) on the 0.75% fibrin microfibers at 1500:37.5:0 ratio (Fig S5). (Step 2) We then cultured hASCs and HUVECs in the absence of NRVCMs to assess vascular assembly on the fibers. We used 37.5 hASCs/mm^2^ to be consistent with the cell densities determined in the NRVCM:hASC co-cultures and varied the concentration of HUVECs (Fig. 5A). The hASCs co-cultured with HUVECs appeared to stain positive for smooth muscle actin (αSMA) in close proximity to endothelial cells (Fig. 5B). Robust vessel assembly was observed on 0.75% and 2.0% fibrin microfiber sheets at the ratios of 0:37.5:75 and 0:37.5:150. Since the NRVCM cultures required a minimum of 6% serum, we tested vascular assembly in the presence of 6% serum. Quantification of vessel length and interconnectivity yielded similar results as for 2% serum (the typical serum concentration for culturing HUVECs) (Fig. 5C,D). Higher magnification images of the cord structures on these fibrin microfibers displayed voids in the cross-sectional view, which indicated lumen formation (Fig. 5E).

(Step 3) In addition to determining suitable hASC and HUVEC concentrations, we assessed postponing the seeding of hASCs and HUVECs unto the confluent NRVCMs to a time later than Day 3. Since the NRVCM only cultures exhibited peak electrophysiological characteristics at Day 14, the tri-culture system was developed in which NRVCMs were seeded at 1500 cells/mm^2^ and cultured for 14 days before hASCs/HUVECs were added (Fig. 6A). The NRVCMs were pre-plated in tissue culture flasks to enrich the cardiomyocyte population. Three groups were studied using the ratios 1500:37.5:37.5, 1500:37.5:75, and 1500:37.5:150 and compared to a NRVCM only (1500:0:0) control. Through CD31 staining, we observed increased vascular cord length and interconnectivity as the concentration of HUVECs increased (Fig. 6B–D).

**Figure 7:**
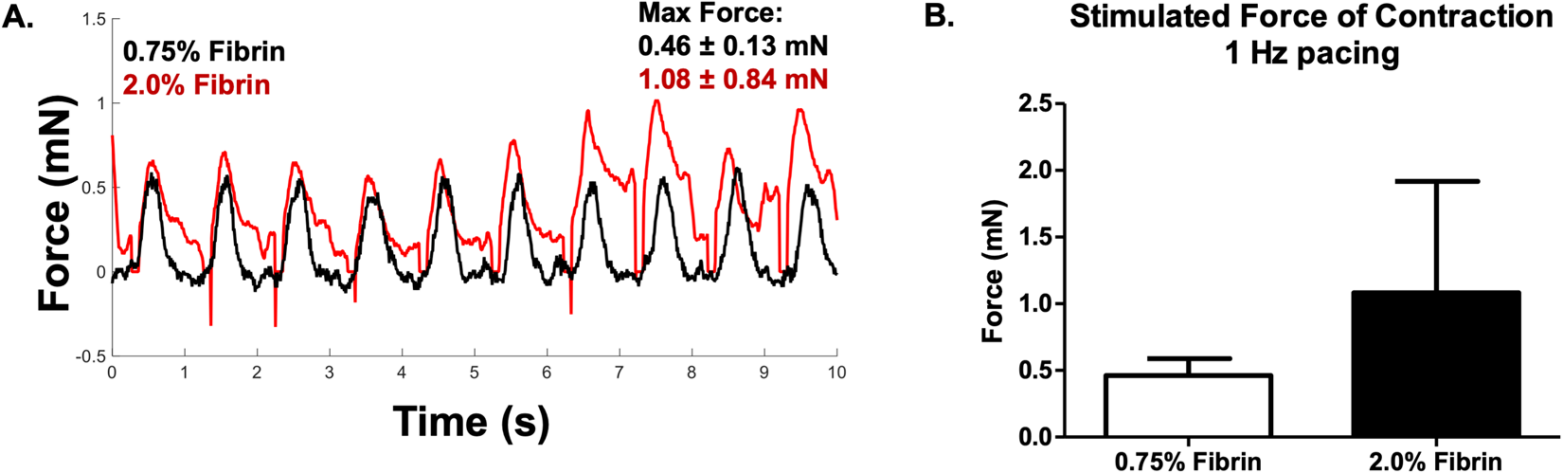
Stimulated Force of Contraction of Engineered Cardiac Tissue with Endothelial Networks. (**A**) Point paced trace of force of contraction of tri-culture patches 0.75% fibrin (black) overlaid with 2.0% fibrin (red) (**B**) Graphical representation of force of contraction measurement of tri-culture system.

After 21 days of culture, constructs were optically mapped. At 1 Hz pacing, there were no significant differences in the tri-culture conduction velocities on the 0.75% or the 2.0% fibrin microfiber sheets compared to their respective cardiomyocyte only controls. Conduction velocities on 0.75% and 2.0% fibrin, were 13 ± 1.3 cm/s (n = 6) and 14 ± 1.5 cm/s (n = 4) respectively for the ratio 1500:37.5:37.5, 15 ± 1.9 cm/s (n = 6) and 13 ± 1.5 cm/s (n = 9) for the ratio 1500:37.5:75, and 14 ± 2.3 cm/s (n = 7) and 14 ± 0.6 cm/s (n = 5) for the ratio 1500:37.5:150 compared to 14 ± 2.1 cm/s (n = 6) and 13 ± 1.0 cm/s (n = 4) for control (Fig. 6E,F). We saw similar trends at other pacing rates (Fig. S6A). We found that there was no statistical difference in action potential duration (both 80% and 30%) between the control groups and the ratio 1500:37.5:150 but the action potentials appeared to be prolonged in the 1500:37.5:37.5 and 1500:37.5:75 groups after 21 days of total culture time on both stiffness sheets (Fig. 6G,H, S6B,C). The control group had maximum capture rates of 3.4 ± 1.3 Hz (n = 6) on 0.75% fibrin and 3.2 ± 0.4 Hz (n = 10) on 2.0% fibrin compared to those of the 1500:37.5:150 group, which achieved higher rates of 3.8 ± 1 Hz (n = 6; 0.75% fibrin) and 3.9 ± 0.7 Hz (n = 5; 2.0% fibrin) (Fig. 6I, Fig. S6D). We tested the stimulated force of contraction of the 1500:37.5:150 groups at 1 Hz pacing and found statistically higher forces on the 2.0% fibrin group (1.1 ± 0.8 mN) compared to the 0.75% fibrin group (0.5 ± 0.1 mN) (Fig. 7).

## Discussion

Cardiac tissue engineering strategies may be used to develop useful biomimetic models of vascularized cardiac tissues for studying cell-cell and cell-biomaterial interactions. In this study, we demonstrated that fibrin microfibers cultured with NRVCMs, HUVECs, and hASCs resulted in the development of a cardio-mimetic tissue that contains endothelial networks with the potential to serve as experimental *in vitro* models. This study assessed the conditions needed to promote cardiomyocyte survival and functionality while promoting vascular assembly. For the first time, we were able to engineer contractile cardiac tissues in direct proximity with stable endothelial tube-like structures on fibrin microfibers. NRVCMs are phenotypically robust and have been widely used to study morphological, electrophysiological, and biochemical characteristics of cardiomyocytes. It is intrinsically advantageous to use these rather than human induced pluripotent stem cell derived cardiomyocytes to evaluate the tri-culture system. However, future studies will incorporate the use of human induced pluripotent stem cell derived cardiomyocytes.

Previous researchers utilized isotropic fibrin hydrogels that relied on cell-generated stresses to create cell alignment^27–29^ to induce the cardiomyocytes to elongate, connect, and form a syncytium. In contrast to fibrin hydrogels, fibrin microfiber sheets displayed tunable mechanical properties, provided topographical alignment cues, and could be sutured onto the myocardium as a proof-of-concept for a cardiac patch^19^. To mimic the mechanical properties of native tissue extracellular matrix during homeostasis and the stiffer state that is present following myocardial infarction, two different concentrations of fibrinogen were used to engineer fibrin microfiber sheets with tensile moduli of 50 kPa and 90 kPa. The adult rat heart tissue ranges from 22 to 50 kPa^30^. The reported tensile modulus for rat myocardium in the long fiber direction is 43 ± 9 kPa and 12 ± 5 kPa in the short myofiber direction^11,13^. In addition, the epicardium increases in stiffness from 12 kPa in neonatal rats to 39 kPa in adult rats^31^. Given these values, we hypothesized that the 50 ± 11 kPa stiffness (measured in the longitudinal direction) was biomimetic (healthy rat heart), while the higher 90 ± 16 kPa stiffness mimicked stiffer pathologic tissue.

Neonatal rat heart isolations may consist of cardiomyocytes, endothelial cells, fibroblasts, and perivascular cells^20^ and fibrin microfiber sheets cultured with NRVCMs-only for up to 2 months may contain trace amounts of non-cardiomyocytes. Electrophysiological properties increased over time up until 28 days and gradually decreased by 2 months of culture. There is a possibility that in this system fibroblasts that remained from the enriched population proliferated in the presence of 6% serum, or differentiated into myofibroblasts on the substrate^32^. The conduction velocity was significantly higher in the 0.75% fibrin group compared to the 2.0% group over time. This finding was consistent with previous reports indicating that cells cultured on softer matrices or near the stiffness of the native myocardium show larger calcium transients, contained more sarcoplasmic calcium stores, and generated more force than when cultured on stiffer matrices^14,15,18,30^. The NRVCMs on the fibrin microfiber sheets took longer (~28 days) to achieve peak electrophysiological properties relative to those grown in monolayer cultures (~7 days)^26^. These differences can likely be attributed to cell-matrix interactions and the use of more compliant substrates which prolonged the rate at which cells would undergo morphogenic changes and form matrix adhesions that are more *in vivo*-like^33^.

The anisotropy ratio of cardiomyocytes cultured on the 0.75% fibrin (~2.2) was more similar to that of the native rat myocardium (2.3)^34^ than those on the 2.0% fibrin microfiber sheets (~1.4). Similarly, cells on the softer of the two substrates exhibited increased force of contraction compared to those on the 2.0% microfiber sheets. The force of contraction on the softer substrate (~2.3 mN) was also higher than on the stiffer microfiber sheets (~1.4 mN). While it appears counter-intuitive that the force of contraction is lower in the stimulated samples, this could potentially be related to negative force frequency relationships noted in rat cardiomyocytes in the frequency range of 0.5 – 2.0 Hz^35^. Future studies will include electrical stimulation during the culture period to promote tissue maturation and increase the forces of contraction^36–38^.

Fibrin microfiber sheets also supported the development of dense vascular structures. We found that in both the 0.75% and 2.0% fibrin microfiber sheets there was robust vascular assembly. Current literature suggests that softer substrates better support HUVEC network formation^39,40^. In our study, we found that vessel assembly occurred on both substrate stiffnesses and that vessel length was relatively independent of cell ratios and serum contents suggesting that the vascular morphogenesis of HUVECs is fairly robust in our cultures^41^. We increased serum concentration for vascular assembly from 2% to 6% since this serum concentration was necessary to maintain cardiomyocyte contractility.

Developing a contractile, electrically responsive, vascularized cardiac patch required several iterations of cell concentration and seeding time points. In initial studies we utilized a protocol optimized for tri-cultures on tissue culture plastic cover slips^26^. We found that culturing cardiomyocytes for 2 days prior to the addition of hASCs and ECs yielded grafts with poor electrophysiological properties although vessel assembly was successful (Fig. 6). NRVCMs on the electrospun fibrin microfiber sheets took longer to elongate and form gap junctions with other cardiomyocytes (~14 days) compared to those cultured on traditional tissue culture plastic (~1–2 days)^42^. To overcome these limitations, it was necessary to increase the NRVCM seeding concentration six-fold, lower the concentration of serum used in the cultures (to mitigate the effects of cardiac fibroblast proliferation), and to reduce the relative ratio of NRVCM:hASCs (20:1) compared to previously reported values^26,43^. Interestingly, while 1,500:37.5 (NRVCM:hASC) cultures resulted in reproducible conduction (CV: 10 cm/s (50 kPa) & 6 cm/s (90 kPa)), the conduction velocities increased further when HUVECs were included in the 1,500:37.5:75 cultures (CV: 15 cm/s (50 kPa) & 13 cm/s (90 kPa)). This compared favorably with NRVCM-only control samples (14 cm/s (50 kPa) & 13 cm/s (90 kPa). We speculate that paracrine factors secreted by endothelial cells could enhance NRVCM-hASC crosstalk leading to better conduction properties^44^. Previous studies have shown that paracrine signaling from vessel networks promote survival of cardiomyocytes and enhanced electrophysiological properties^45^. In addition, hASCs have been shown to modulate the matrix by secreting collagen and have the potential to act as a mechanical support or bridge between cells^46,47^. The similar characteristics observed between the 0.75% and 2.0% fibrin microfiber sheets could likely be attributed to the modified microenvironment developed by the supporting cells. In future studies, we will assess the potential for replicating this tri-culture system using human cells all derived from induced pluripotent stem cells.

## Conclusions

We have successfully generated a electromechanically coupled biomimetic cardiac tissue that is composed of contractile cardiomyocytes and vessel networks. To achieve this cardiac tissue, we employed uniaxially aligned fibrin microfiber sheets with biomimetic stiffnesses and developed a protocol for successfully co-culturing NRVCMs, hASCs, and HUVECs. These studies confirmed previous reports that NRVCM physiology is enhanced on softer substrates (50 kPa vs. 90 kPa), but also provided novel insight in demonstrating that the effect of substrate stiffness on cardiomyocyte function is not absolute but is also influenced by heterotypic cell-cell interactions. Upon finding a concentration of hASCs that would not negatively impact electrophysiology but maintain robust vascular development, we were able to develop a vascularized cardiac system. Ultimately, tri-cultures on the two substrates yielded identical electrophysiological and vessel network characteristics *in vitro* though cells on the stiffer substrates generated higher contractile forces in response to electrical stimulation. These studies indicate that fibrin microfiber sheets are a potentially viable source for engineering functional, vascularized cardiac tissue.

## Materials & Methods

### Fibrin Microfiber Sheet Production

In brief, fibrin microfibers were developed by electrospinning fibrinogen from a syringe tip under an applied electric field of 5 kV onto a rotating stage containing thrombin and calcium chloride for crosslinking (Fig. 1A,D). To ensure similar quantities of fibrin in each group, the solutions were electrospun onto the collector for 17 minutes when using 2.0 wt% fibrinogen or 20 minutes total using 0.75 wt% fibrinogen with a pause in spinning after 10 minutes to refill the 1 cc syringe. Upon completion of the spin time, the fibrin sheets were allowed, at minimum, two additional minutes in the crosslinking solution to ensure fibrinogen deposited later in the spin had sufficient time to stabilize. A 1 cm × 1 cm plastic frame was then placed onto the resulting fibrin sheet and flipped end over end a total of six times, being careful to ensure entrapped air bubbles did not disrupt alignment. Once collected with the frames, fibrin sheets were placed in deionized (DI) water to dilute residual thrombin and were stored in DI at 4 °C. Prior to cell seeding, excess water was wicked away from the fibrin sheets to increase adhesion of cardiomyocytes. Partially dried fibrin sheets were used within a couple days to prevent damage to fibrin sheets due to dehydration.

### Scanning Electron Microscopy (SEM)

SEM images for the native tissue samples and hydrogel microfibers sheets (with or without cells) were acquired using an SEI Quanta 200 Environmental SEM. All samples were fixed with cold methanol and then thoroughly washed with Dulbecco’s Phosphate Buffer Saline (5× wash, 30 minutes each at room temperature). Then, the samples underwent gradual ethanol dehydration (25%, 50%, 75% 100%), after which they were critical point dried using a Tousimis Samdri-795 and sputtered with 4 nm of palladium layer using an Anatech USA Hummer 6.2 prior to imaging

### Mechanical Testing

Fiber stiffness experiments were performed using a custom bioreactor^48^. Bulk cylindrical fibers, (3 cm × 1 mm) were clamped between the actuator and the force sensor in the bioreactor system allowing for an active region of approximately 18 mm between the clamped regions. The samples remained hydrated throughout the duration of the testing period. The elastic modulus was determined through fitting the linear region of the resulting stress stain curve of extension of the fiber to 100 percent strain at 1 percent strain/ second and recording the corresponding force with the force sensor (Cooper Instruments) attached to a strain gauge amplifier unit (Industrologic SGAU). The voltage output from this system was read through the Lab Jack U3-HV and was recorded in real time through Lab Jack’s custom software. Following the tensile test, the data was converted to stress by using the measured diameter of the fiber through bright-field imaging prior to mechanical testing.

### NRVCM Culture on Fibrin Microfiber Sheets

All animal procedures were performed in compliance with guidelines set by the Johns Hopkins Committee on Animal Care and Use and all federal and state laws and regulations following their review and approval of the experimental protocols. NRVCMs were seeded at a concentration of 1.5 × 106 cells/cm2 in 80 μL of NRVCM media containing 10% FBS onto 1 cm × 1 cm fibrin microfibers sheets. Fibrin microfiber sheets were placed onto agarose-coated culture plates to prevent cells from attaching to tissue culture plastic. Upon initial seeding, cells were allowed to attach for 1 hour before adding more media for culture. Cells were fed through Day 2 with 3 mL of NRVCM media with 10% fetal bovine serum (FBS) and 33 μg/mL Aprotinin, antifibrinolytic molecule to preserve the fiber (USB Affymetrix). On day 3 of culture, microfibers were washed with Dulbecco’s Phosphate Buffered Saline (DPBS) and fed NRVCM media with 6% FBS and 33 μg/mL Aprotinin (USB Affymetrix). Media was changed every other day until electrophysiological and functional testing occurred.

### Tri-Culture of NRVCM:hASC:HUVEC Fibrin Microfiber Sheets

Human Adipose Derived Stem Cells (hASCs) isolation was performed after obtaining informed consent from donors at the Johns Hopkins University, under an Institutional Review Board approved protocol according to published methods. We utilize the nomenclature listed below to describe the cell-plating densities throughout the manuscript. We report thousands of cells per 1 cm^2^ fibrin microfiber sheet for co-cultures and NRVCM:hASC:HUVEC tri-cultures. Therefore, for NRVCM:hASC co-cultures, the HUVEC number is reported as zero. Likewise, in hASC:HUVEC experiments, the NRVCM numbers are reported as zero. The details of seeding are presented in Supplementary materials. In brief, cardiomyocytes were seeded on fibrin microfibers as described above and this number is always reported as 1500. After two weeks of culture, hASCs and HUVECs (1500:37.5:37.5; 1500:37.5:75; 1500:37.5:150) were seeded on the surface of cardiomyocytes and fed tri-culture media until analysis was completed.

### Optical Mapping & Analysis

Optical mapping measured changes in transmembrane voltage of electrically conductive cells and provided visual patterns of excitation and repolarization^49^. Fibrin fibers were stained with 10 μM di-4-ANEPPS (Invitrogen), a voltage sensitive dye, in Tyrode’s solution for 10 minutes at 37 °C while being protected from light^49–52^. After staining, the dye solution was removed and cells were then washed with Tyrode’s solution. The cells were then placed on a heated stage in Tyrode’s solution with 5 μM blebbistatin to prevent motion artifacts. Cells were electrically stimulated by placing an electrode near the edge of the coverslip, out of the field of view of the camera, and initially paced at 0.5 Hz and incrementally increased by 0.5 Hz until the sample no longer captured the paced cycle length or had reentrant spiral waves. At each new cycle length, fibrin fibers were allowed to be stimulated for 1 minute to achieve steady state between each new pacing rate. Optical mapping was performed using a CMOS camera (MiCAM Ultima).

### Measuring Forces of Contraction

Force of contraction experiments were performed using the custom bioreactor^48^ described above. Following the indicated culture period and conditions, longer microfiber sheets (3 cm × 1 cm) were clamped between the actuator and the force sensor in the bioreactor system allowing for an active region of approximately 18 mm between the clamped regions. Following loading of the sample between the clamps, the bioreactor was placed in an incubator for approximately 15 min to ensure that the sample resumed contraction. Measurements of the forces of contraction of contractile samples were recorded by the force sensor (Cooper Instruments 50 gram max load cell) attached to a strain gauge amplifier unit (Industrologic SGAU). The spontaneous forces of contraction were recorded for several minutes. Following these recordings, a custom-built stainless steel and platinum point electrode was placed such that it contacted the sample along the outer edge half way along the length of the sample. The sample was stimulated with 10 volts of direct current with a 20 ms pulse width at a variety of frequencies until it was no longer able to keep up with the stimulus. The force-length relationship data was measured at a frequency of 1 Hz. The sample was extended in intervals of 2% of the initial loading length, and the stimulated (1 Hz, 10 V) contractions were recorded at each length up to 20% strain.

### Immunocytochemistry

Fixed samples were permeabilized with 0.2% Triton X-100 in DPBS with 5 mM HEPES for 10 minutes and blocked with 10% normal goat serum (Sigma Aldrich) or normal donkey serum (Sigma Aldrich) for 3 hours at room temperature. The samples were incubated overnight with primary antibodies diluted in DPBS with 5 mM HEPES with 10% normal goat serum or normal mouse serum at 4 °C. Primary antibodies were incubated for overnight at 4 °C and washed three times with DPBS after incubation. Samples were incubated with fluorescently tagged secondary antibodies at 4 °C overnight. Samples were washed three times with DPBS. Samples were incubated with DAPI (1:2000) (Sigma Aldrich) diluted in DPBS. Coverslips were mounted with mounting medium (1:1 Glycerol:DPBS) and imaged using either a Zeiss Axio Observer inverted fluorescence microscope or Zeiss LSM 510 confocal microscope through 5×, 20×, 40×, or 63× objectives. Immunostained images were z-projected and a uniform threshold was applied to all images. Images were subsequently analyzed with AngioQuant software to determine the total vessel length, area, and interconnectivity.

### Statistical Analyses

All experiments were conducted with a minimum of three biological replicates. Quantitative data are expressed as the mean ± standard deviation. Statistical analysis was performed using GraphPad Prism 5/6 software. Statistical significance was determined by two-way ANOVA with Bonferroni post-hoc test or t-test and is denoted as * p < 0.05, **p < 0.01, ***p < 0.001.

### Ethical Approval

All animal procedures were performed in compliance with guidelines set by the Johns Hopkins Committee on Animal Care and Use and all federal and state laws and regulations. Human Adipose Derived Stem Cells (hASCs) isolation was performed at the Johns Hopkins University, under an Institutional Review Board approved protocol according to published methods.

## Supplementary information

Supplementary Information.

## Data Availability

The raw/processed data required to reproduce these findings cannot be shared at this time due to technical limitations.
