# Supplementary material for: Biomimetic Model of Contractile Cardiac Tissue with Endothelial Networks Stabilized by Adipose-Derived Stromal/Stem Cells
Source: Sci Rep. 2020 May 20;10:8387. doi: 10.1038/s41598-020-65064-3 (PMC7239907; doi:10.1038/s41598-020-65064-3)
Supplement: Supplementary file 1 — Supplementary Information. [file 41598_2020_65064_MOESM1_ESM.docx]

**Biomimetic Model of Contractile Cardiac Tissue with Endothelial Networks Stabilized by Adipose-Derived Stromal/Stem Cells**

Justin Morrissette-McAlmon^1,2^, Brian Ginn^1,3^ , Sarah Somers^1,2^ , Takuma Fukunishi^5^, Chanon Thanitcul^2^, Alexandra Rindone^1,2^, Narutoshi Hibino^5^, Leslie Tung^2^, Hai-Quan Mao^1-4^, Warren Grayson^1-4*^

**SUPPLEMENTARY METHODS**

**S2.1 Isolation of Neonatal Rat Ventricular Cardiomyocytes (NRVCs)**

All animal procedures were performed in compliance with guidelines set by the Johns Hopkins Committee on Animal Care and Use and all federal and state laws and regulations. In brief Neonatal Rat Ventricular Cardiomyocytes (NRVCMs) were enzymatically dissociated from the hearts of 1-3 day old Sprague Dawley rats (Charles River Laboratories, Frederick, MD) with the use of 0.25mg/mL trypsin (US Biochemicals) and 1mg/mL collagenase (Worthington). Freshly isolated cells were resuspended in Medium 199 culture medium (Life Technologies) supplemented with 10% heat inactivated fetal bovine serum (Sigma Aldrich), L-glutamine, glucose, penicillin, vitamin B12, HEPES buffer, and MEM non-essential amino acids. Cells are pre-plated in a flask to reduce the number of fibroblast and enrich the cardiomyocyte population. The cardiomyocyte population is nearly 70 ± 6% (n=4) post pre-plating after 7 days of culture in monolayer (**Fig S4)**.

**S2.2 Isolation of Adipose Derived Stem/Stromal Cells:**

Human Adipose Derived Stem Cells (hASCs) isolation was performed at the Johns Hopkins University, under an Institutional Review Board approved protocol according to published methods. Briefly, fresh human subcutaneous adipose lipoaspirates were obtained under informed consent from healthy donors undergoing elective liposuction. The lipoaspirate tissue was extensively washed with warm phosphate buffer saline solution to remove erythrocytes and then digested in PBS supplemented with 0.1% Collagenase Type I (Worthington), 1% BSA, and 2 mM CaCl2 for one hour at 37°C. Following room temperature centrifugation at 300G and resuspended in Stromal Vascular Fraction Medium (DMEM/F-12 (Life Technologies) supplemented with 10% FBS (Atlanta Biologicals) and 1% penicillin/streptomycin), the stromal vascular pellet obtained from 35mL of lipoaspirate digest was plated in a T175 flask (0.2mL per cm2). After 24 hours of incubation at 37°C, 5% CO2, the adherent cells were washed with warm PBS and maintained in Stromal Medium until 80-90% confluent. The adherent population (“passage 0”) was harvested by digestion with trypsin (0.05%)/EDTA(1mM) at 37°C for five minutes, washed with Stromal Medium and cryopreserved. Prior studies have shown that there are no deleterious effects on hASCs due to cryopreservation, such as loss of viability or multipotency. For expansion, hASCs were thawed and cultured in expansion medium which includes DMEM high glucose (Life Technologies), 10% FBS (Atlanta Biologicals), 0.5%penicillin/streptomycin (Cellgro), 0.5%antibiotic/antimycotic, 1ng/mL FGF2 (PeproTech). Cells were utilized at passage 2-3.

**S2.3 Human Umbilical Vein Endothelial Cell Culture:**

A vial of pooled Human Umbilical Vein Endothelial Cells (HUVECs) (Lonza, USA) were expanded in tissue culture flasks up to passage 4-5 using Endothelial Basal Medium 2 (EBM-2) culture medium (Lonza, USA) supplemented with the Endothelial Growth Medium-2 (EGM-2) Bullet Kit (Hydrocortisone, human fibroblast growth factor (hFGF)-2, vascular endothelial growth factor (VEGF), R3-insulin-lke growth factor (IGF)-1, ascorbic acid, human epidermal growth factor (hEGF), Gentamicin & Amphotericin (GA-1000), and Heparin) and 2% FBS. HUVECs were used for experimentation at Passage 4-6.

**S2.4 hASC & HUVEC Culture on Fibrin Microfiber Sheets:**

hASCs and HUVECs were mixed together in ratios of 0:100:100, 0:100:50, 0:100:20, 0:100:10, 0 and seeded in 80μL of media onto fibrin microfiber sheets (1cm^2^), which were placed onto agarose coated culture plates to prevent cells from attached to tissue culture plastic. The constructs were cultured *in vitro* for 7 days and fed every other day with EGM-2 (Lonza). To quantify the vessels, Z-stack projections were analyzed using AngioQuant software.

**S2.5 Tri-Culture of NRVCM:hASC:HUVEC Fibrin Microfiber Sheets:**

NRVCs were seeded at a concentration of 1.5 × 10^6^ cells/cm^2^ in 80μL of NRVCM media containing 10% FBS onto fibrin microfiber sheets (1cm^2^). The fibrin microfiber sheets were placed onto agarose coated culture plates to prevent cells from attached to tissue culture plastic. Cells were fed 3mLs of NRVCM media containing 10% FBS and 33μg/mL Aprotinin (USB Affymetrix). On the next day microfibers were washed with DPBS and fed with NRVCM media containing 10% and 33μg/mL Aprotinin (USB Affymetrix). On day 3 of culture, microfibers were washed with DPBS and feed NRVCM medium containing 6% FBS and 33μg/mL Aprotinin (USB Affymetrix). Media was changed every other day until day 14. On day 14, media was removed and hASCs and HUVECs were added at specific concentration (i.e. 3.7 × 10^4^ hASC/cm^2^ and 1.5 × 10^5^HUVEC/cm^2^) in 20μL of tri-culture media. After one hour of incubation, cells were fed 3mL tri-culture media containing 33μg/mL Aprotinin, which is EGM-2 Media with 6% HI-FBS and 20μM Glucose. Media was refreshed every other day until 21 days of culture. At 21 days, microfiber sheets underwent functional testing, which consisted of optical mapping and force of contraction. Upon harvest, cells were assessed using optical mapping and were fixed with ice-cold methanol overnight at -20C for immunocytochemistry analysis.

**S2.6 Viability Assessment**

To determine cell viability on fibrin μfibers, the microfiber sheets were removed from frames and placed into 500µL Deoxyribonucleic Acid (DNA) lysing solution (10mM Tris, 1mM EDTA, 0.1% Triton X-100, and 0.1 mg/mL proteinase K) and frozen at -20**°**C. Samples were subsequently heated at 50 **°**C overnight to digest proteins and inactivate nucleases. PicoGreen dsDNA quantification assay was carried out according to the manufacturer’s instructions. Briefly, the samples were combined with PicoGreen reagent (100 µL sample + 100 µL PicoGreen) and transferred (200 µL) into a black 96-well plate along with DNA standards. Fluorescence was measured at excitation 485nm/ emission 528 nm using a fluorescent plate reader. Sampling days included day 0, day 1, day 7, and day 14. Live/Dead viability assay assesses cell viability using calcein AM. Calcein AM is a cytoplasmic dye cleaved by cytoplasmic esterases that ultimately stains cytoplasm green was used at a concentration of 5μM. Ethidium Bromide is a nuclear stain that only penetrates dying or dead cells and emits red fluorescence was used at a concentration of 2μM. Both dyes were mixed in NRVCM media and incubated at 37°C for 20 minutes and washed with DPBS. Cells were imaged on the LSM-510 confocal.

**S2.7 Primary and secondary antibodies.**

We used the following primary antibodies mouse anti-Sarcomeric α-Actinin [1:200] (Sigma Aldrich), rabbit anti-Connexin-43 [1:100] (Sigma Aldrich), and Cy3 conjugated mouse anti- α Smooth Muscle Actin [1:400] (Sigma Aldrich) or mouse anti-Vimentin [1:200] (Dako)), or mouse anti-CD31 [1:250] (Sigma Aldrich). For secondary antibodies, we used DyLight 488-conjugated goat anti-mouse [1:200] and DyLight 649-conjugated goat anti-rabbit [1:200] (Jackson ImmunoResearch) or corresponding donkey secondary antibodies.

**SUPPLEMENTARY RESULTS**


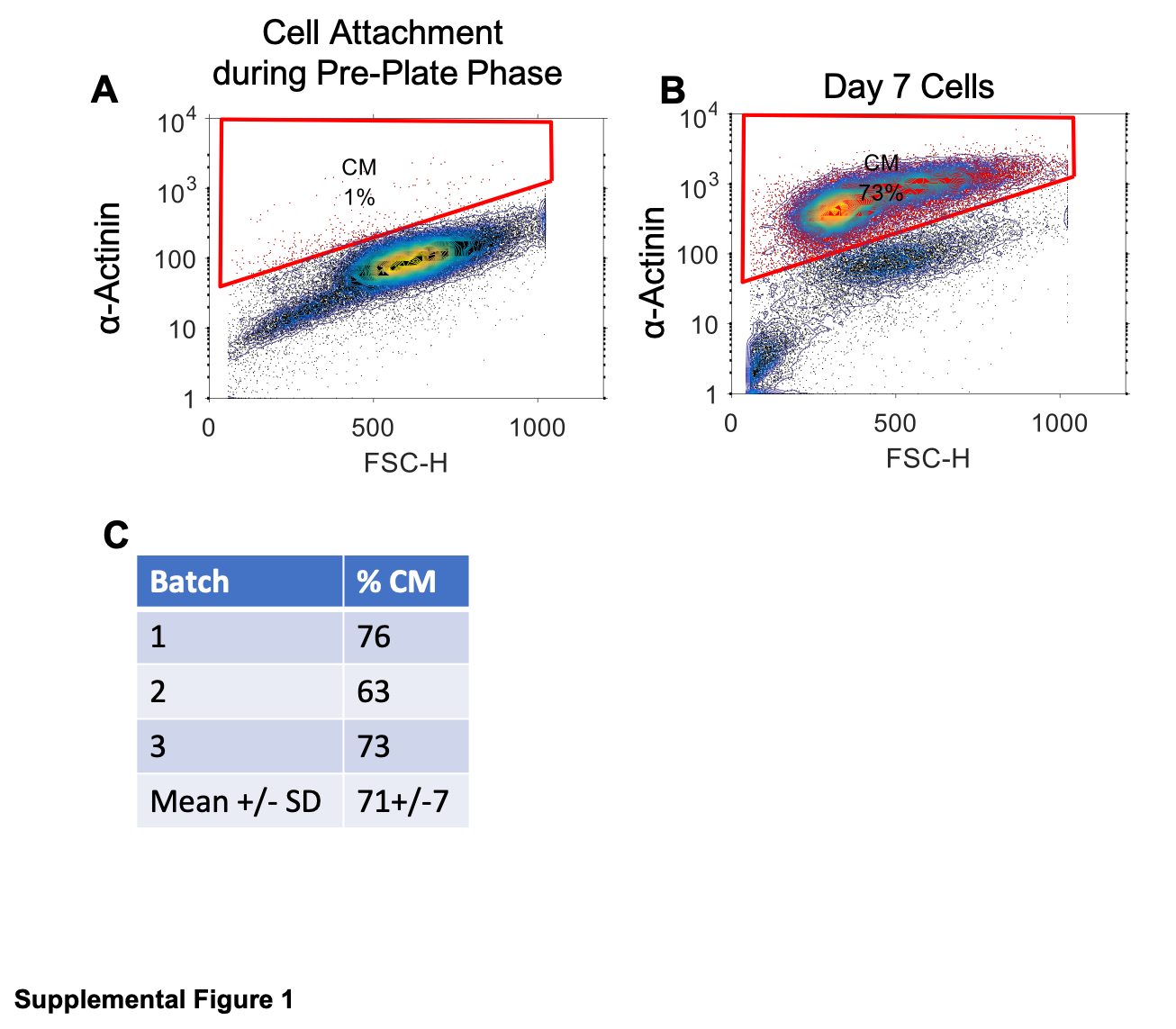


**Supplemental Figure 1**: **Flow cytometry.** Flow cytometry was conducted measuring α-actinin versus forward scatter (FSC-H). **A)** Cells that attached during preplating, which should be non-myocytes, were used as a negative control. **B)** After 7 days in culture, flow cytometry could be used to show two clear groups, one similar to the preplate-attached cells, and one with increased α-actinin expression taken to be cardiomyocytes (denoted by red box). The cardiomyocyte group comprised 73% of the cells. **C)** Table showing the cardiomyocyte fraction across three batches.


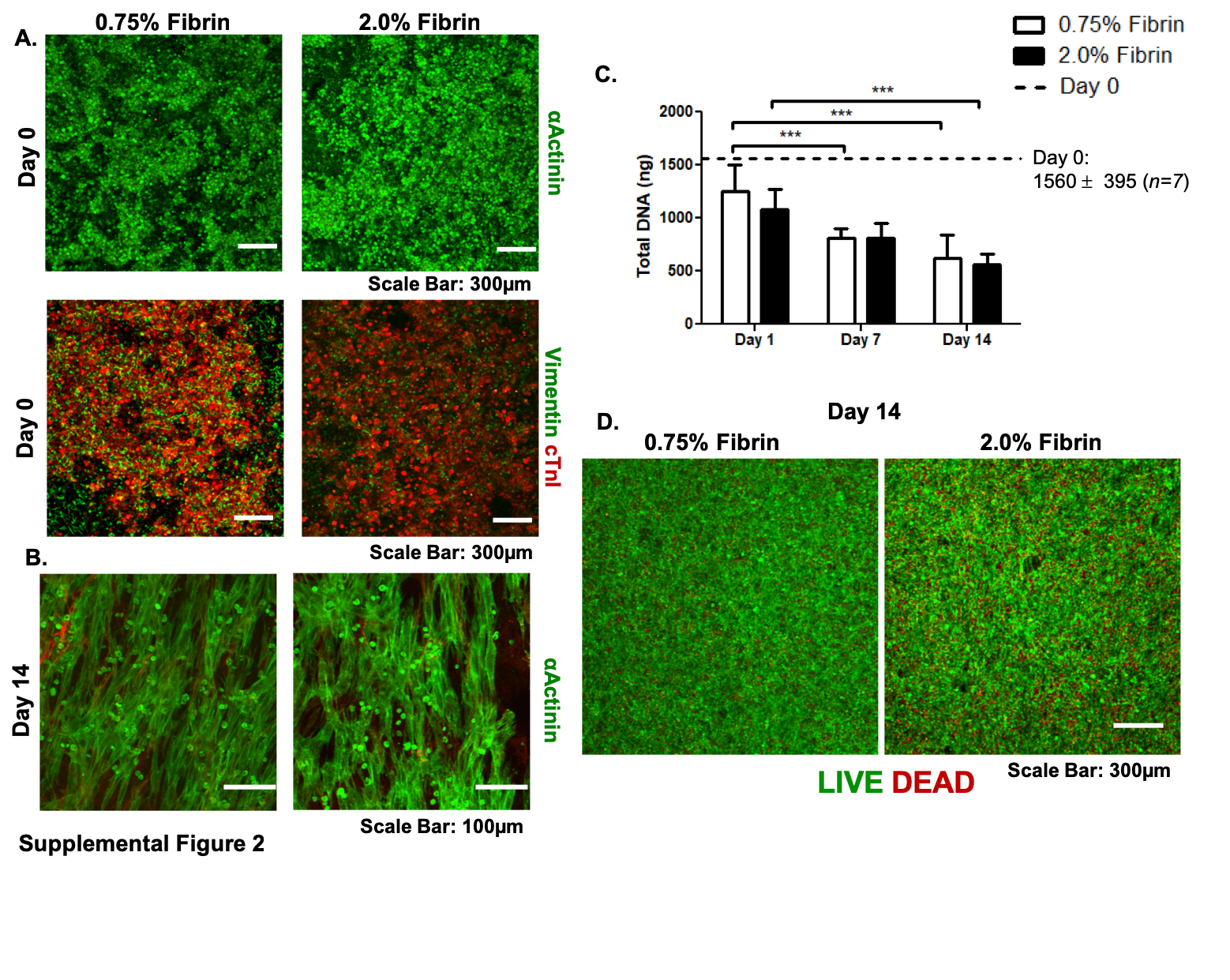


**Supplemental Figure 2: Survival of cardiomyocytes on fibrin microfiber sheets*.* A)** Immunofluorescent staining of cardiomyocytes on fibrin microfibers at Day 0 alpha Actinin (cardiomyocytes) in green on top panel and immediately below vimentin (fibroblast) in green and cardiac troponin I (cardiomyocytes) in red. **B)** Immunofluorescent staining of 0.75% and 2.0% fibrin microfiber sheets **C)** DNA quantification of cardiomyocytes on fibrin microfiber sheets (0.75% and 2.0%) over 14 days relative to Day 0. **D)** Representative images of calcein AM and ethydium bromide stained fibers to qualitatively examine cardiomyocyte survival. ***p < 0.001


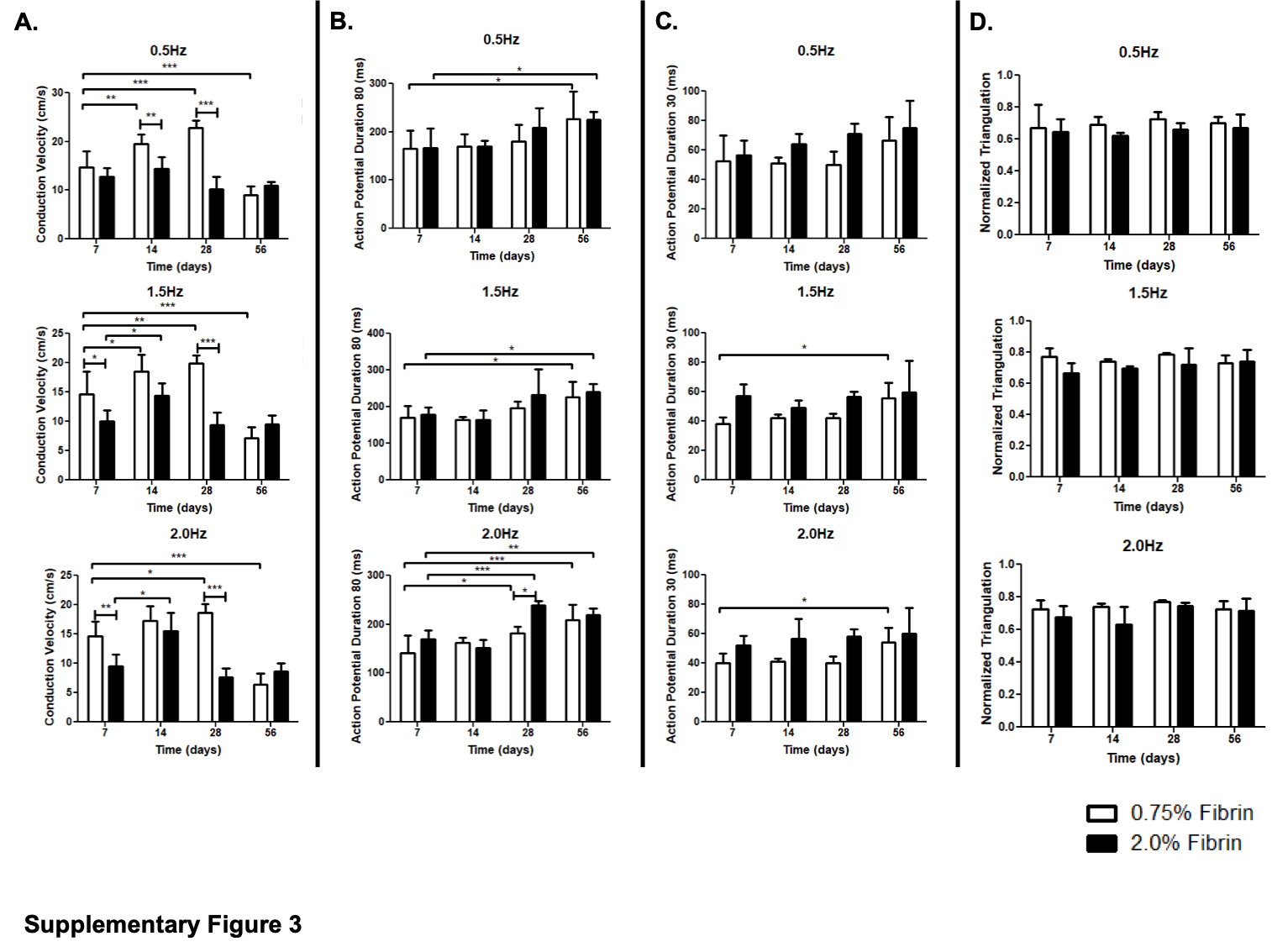


**Supplementary Figure 3:**  **Electrophysiological characterization of cardiomyocytes at 0.5, 1.5, and 2.0 Hz Pacing A)** Conduction velocity of both 0.75% and 2.0% fibrin over time **B)** Action potential duration 80 of both 0.75% and 2.0% fibrin over time **C)** Action potential duration 30 of both 0.75% and 2.0% fibrin over time **D)** Normalized triangulation over time.


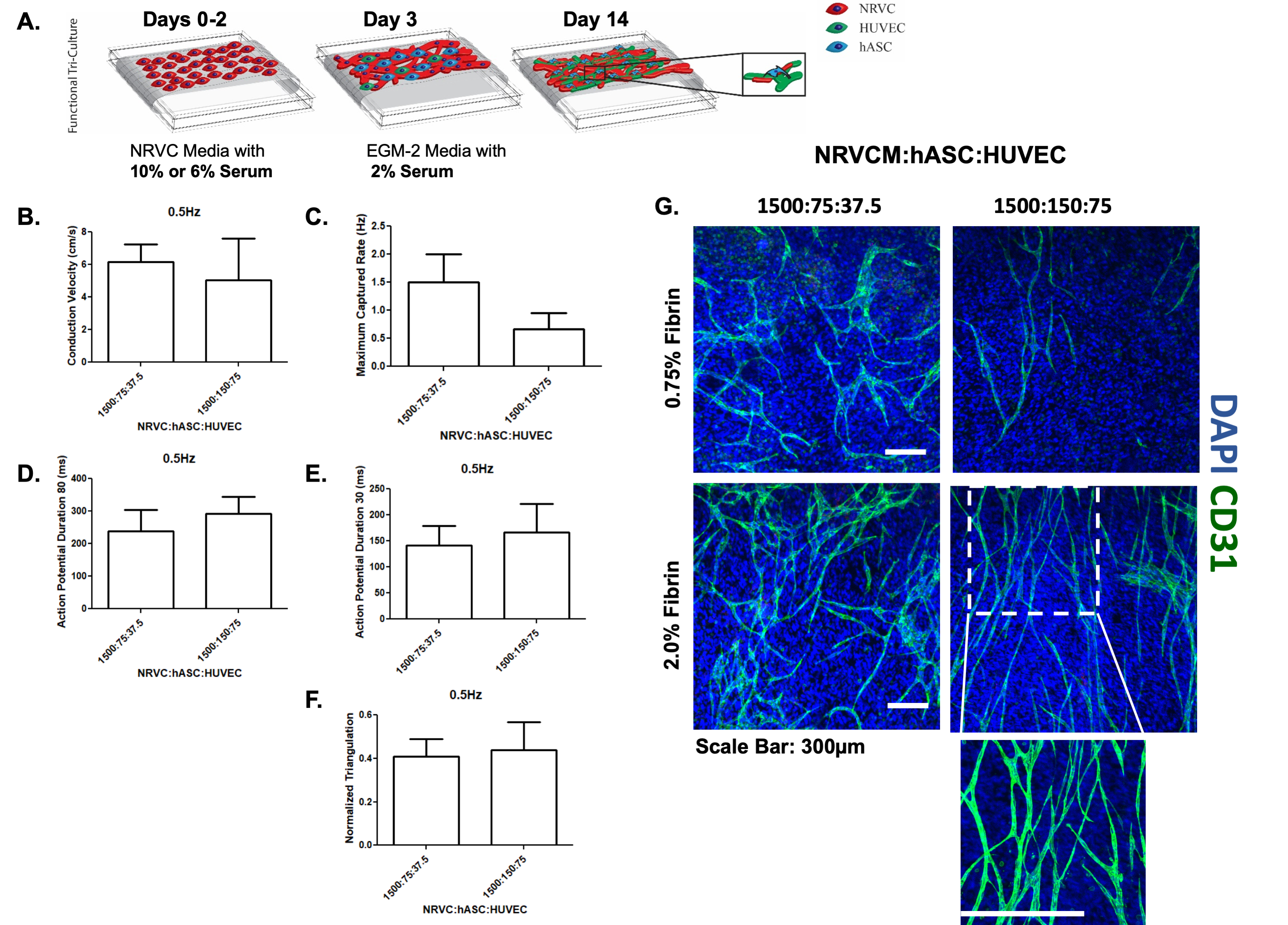


**Supplemental Figure 4: Development of functional tri-culture**. **A)** Schematic showing the additional seeding of supporting cells types after 2 days of CM culture. **B)** Conduction Velocity of tri-culture **C)** Maximum captured rate **D**) Action Potential Duration 80 **E)** Action Potential Duration 30 **F)** Normalized Triangulation **G)** Immunofluorescent staining of CD31/PECAM-1 vessel structures.


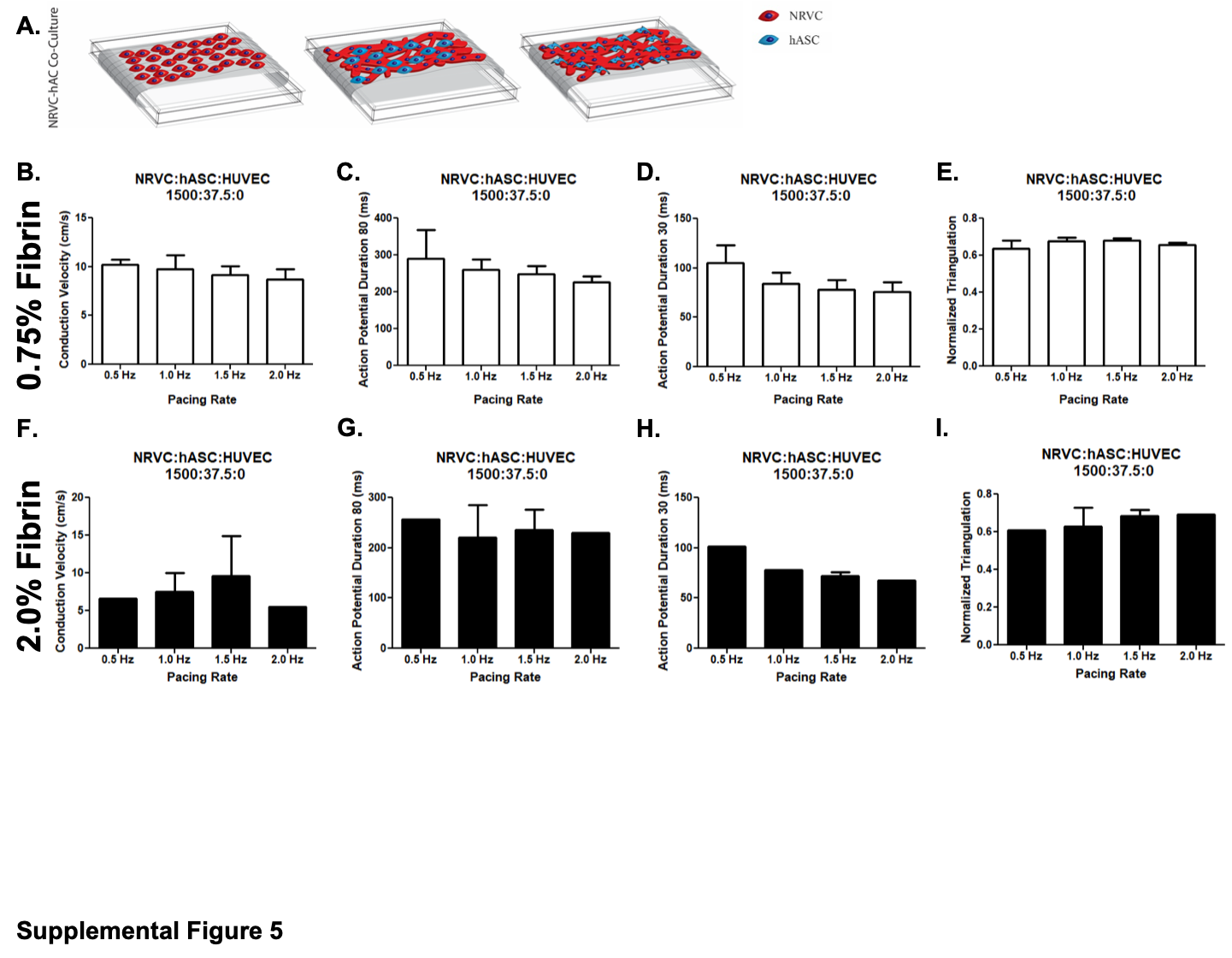


**Supplemental Figure 5: Cardiomyocyte and hASC co-cultures on fibrin microfiber sheets*.*** **A)** Schematic of sequential seeding of hASCs after cardiomyocytes. 0.75% fibrin μfibers at several pacing rates **B)** Conduction velocity **C)** Action potential duration 80 **D)** Action potential duration 30 **E)** Normalized Triangulation. 2.0% fibrin μfibers at several pacing rates **F)** Conduction velocity **G)** Action potential duration 80% **H)** Action potential duration 30% **I)** Normalized Triangulation


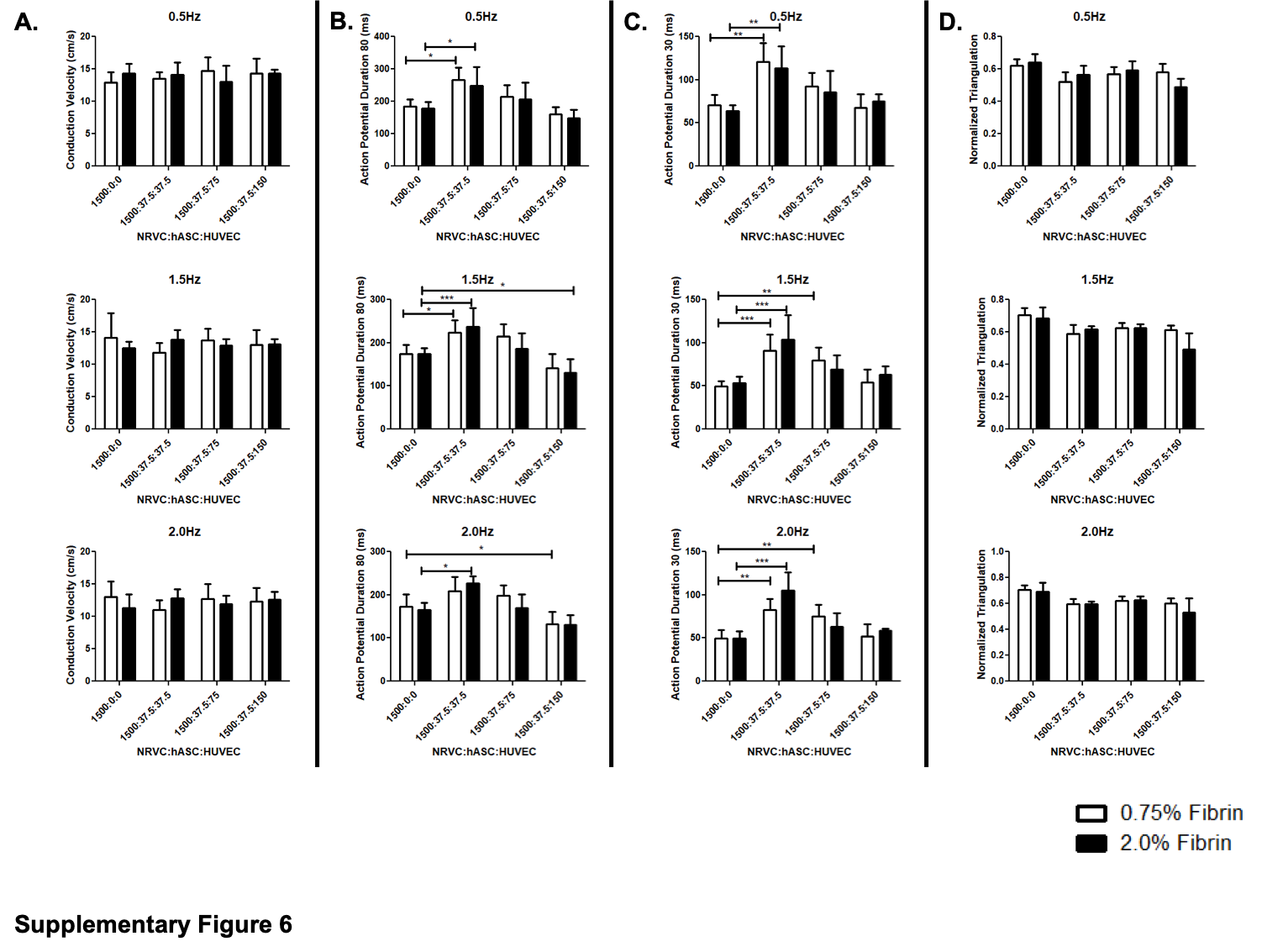


**Supplementary Figure 6:**  **Electrophysiological characterization of tri-cultures grown on** 0.75% and 2.0% fibrin **microfiber sheets with different cell ratios and paced at 0.5, 1.5, and 2.0 Hz. A)** Conduction velocities. **B)**  Action potential duration 80. **C)** Action potential duration 30. **D)** Normalized triangulation.
